# Knowledge and perceptions of pharmacists’ readiness during coronavirus pandemic: the case of United Arab Emirates

**DOI:** 10.1186/s40545-021-00382-z

**Published:** 2021-12-02

**Authors:** Iman A. Basheti, Nizar M. Mhaidat, Sayer Al-Azzam, Rajaa Alqudah, Razan Nassar, Hamzah Alzubaidi, Mahmoud S. Abu-Samak, Eman Abu-Gharbieh

**Affiliations:** 1 Clinical Pharmacy, Department of Clinical Pharmacy and Therapeutics, Faculty of Pharmacy, Applied Science Private University, Amman, Jordan; 2 Department of Clinical Pharmacy, Faculty of Pharmacy, Jordan University of Science and Technology, Irbid, Jordan; 3 Department of Pharmacy Practice and Pharmacotherapeutics, College of Pharmacy, University of Sharjah, Sharjah, United Arab Emirates; 4 Department of Clinical Sciences, College of Medicine, University of Sharjah, 27272 Sharjah, United Arab Emirates

**Keywords:** Coronavirus, Pandemics, Pharmacists, United Arab Emirates

## Abstract

**Background:**

With the outbreak of Coronavirus infection (COVID-19), pharmacists play an important role in supporting local health during this emergency.

**Aim:**

To assess the knowledge and to identify information sources regarding COVID-19 used by pharmacists, to investigate the active and public perceived roles of pharmacists, to explore the role of the pharmacy facilities and health authorities, and to identify barriers that would hinder pharmacists from performing their duties optimally in the United Arab Emirates.

**Methods:**

This descriptive cross-sectional online study was conducted in the UAE during the COVID-19 outbreak, from 18 May to 20 June 2020. A validated online questionnaire addressing participants’ current knowledge about pandemics and COVID-19, source of information, and their perspectives of their role was used. Participants were licensed pharmacists practising in community and hospital pharmacies in UAE, academics, and pharmacy students.

**Results:**

Almost two-thirds of the participants (71.2%) were aged 18–30 years, with 76.2% females. Only 57.5% of participants believed that they got enough education about pandemics, and 88.3% of them followed on the latest coronavirus updates regarding treatments, and that is mainly from the World Health Organization reports (53.9%), followed by health authorities (44.8%). Two-thirds of participants (69.7%) had good/very good current knowledge regarding COVID-19. Knowledge of pharmacy students compared to pharmacists was significantly higher (*p* < 0.001).

**Conclusion:**

The majority of pharmacists and pharmacy students reported that they have a major role in managing pandemics executed through the community pharmacies and that it is their role to ensure the availability of key medications. Policymakers and health authorities are called upon to train pharmacists in advance of emerging situations, supporting and helping them to optimally fulfill their role.

## Background

An epidemic is a disease that affects an enormous number of individuals simultaneously within a population or region. The World Health Organization (WHO) further identifies the epidemic as a disease confined to a district or community [1]. An epidemic becomes a pandemic when the disease spreads over a large area, such as several countries, continents, or even worldwide. The WHO further identifies the pandemic as a universal spread of a new disease. In March 2020, the WHO declared the coronavirus outbreak as a worldwide pandemic. With reference to the past, many respiratory infectious diseases were caused by coronaviruses, such as common cold escalating to the more severe ones, such as the Severe Acute Respiratory Syndrome (SARS) and Middle East Respiratory Syndrome (MERS) [2]. The most lately discovered one is the COVID-19 [3].

COVID-19 is a very contagious virus that spreads rapidly, starting from Wuhan, China, to almost every country worldwide [4]. Several symptoms are associated with COVID-19 infection, such as fever, dry cough, nasal congestion, malaise, headache, muscle pain, loss of taste and/or smell, diarrhoea and vomiting [4].

Implementing infection control measures and public awareness campaigns are necessary to mitigate the spread of the disease [4]. This can be achieved by cleaning hands regularly, keeping a physical distance of at least one meter (3 feet) from others, even if they do not appear to be sick, wearing facial masks and gloves, avoiding touching eyes, nose and mouth, and staying informed regarding the disease [5].

By the end of October 2021, COVID-19 had infected more than 243 million individuals and caused more than four million deaths [6]. Also, within the same period, and specifically in the United Arab Emirates (UAE), more than 739 thousand individuals have been infected, and more than 2000 individuals died [7].

Since the spread of COVID-19 in the UAE (March 2020), the government has handled the situation in a well and organized manner. The government adopted numerous new practices to keep up the performance of all sectors in battling the spread of COVID-19 optimal; for example, hospitals and other health institutions were provided with the support needed to manage all cases of COVID-10, imposing a quarantine on the suspected patient, preparing and providing a medical guide to several healthcare sectors including the private and governmental health facilities, in addition to educational and tourists’ facilities. In addition to executing media and health awareness campaigns emphasizing the importance of hygiene and supplies disinfection, preventive practices to reduce the spread of COVID-19 (i.e., cancelling all events that may include human gatherings) were also implemented [8, 9].

As healthcare experts, pharmacists can take on a significant role in national and community areas to hinder COVID-19 spread. Pharmacists should keep updated information regarding COVID-19 from trusted sources [10]. In addition, pharmacy facilities and health authorities should implement strategies to prepare future pharmacists to deal with pandemics, such as COVID-19 [11]. The burden of responsibility increases, since many barriers would impede the pharmacists from performing their role, such as fear of acquiring the infection [11].

The scope of pharmacy-based services differs between developed and developing countries. In the UAE, pharmacists typically provide basic pharmacy services, such as dispensing prescribed medications, providing over-the-counter and non-pharmaceutical products, and offering self-care advice, whereas enhanced professional services are not provided to a large extent in most pharmacies. However, the findings of a recent study indicated that community pharmacists are enthusiastic and are willing to engage in enhanced services, such as screening, chronic disease management, and medication use reviews with sufficient training and support [12].

Therefore, this study was conducted in UAE to (1) assess the knowledge and identify information sources regarding COVID-19 among pharmacists/pharmacy students, (2) investigate the role of the pharmacists in this area, (3) explore the role of the pharmacy facilities, (4) identify the role of the health authorities in managing COVID-19, (5) and identify barriers that would hinder pharmacists from performing their duties optimally.

## Methods

### Study design and participants

The study objectives were addressed in a descriptive cross-sectional study. This study was conducted in the United Arab Emirates (UAE) during the coronavirus outbreak from 18 May to 20 June 2020. Eligible participants were licensed pharmacists practising in community and hospital pharmacies in UAE, pharmacists working in academic settings, and pharmacy students (first to fifth year). Ethics approval for the study was obtained from the Research Ethics Committee, University of Sharjah (REC-20-05-10-01). Participation in the study did not pose any risk to participants and was voluntary. Potential participants who completed the survey provided informed consent before study participation.

### Survey development

Following an extensive review of the literature, the research team (five professors and one assistant professor in clinical pharmacy and therapeutics, and two pharmacists with a masters in clinical pharmacy degree) put together certain questions to answer the aim and the objectives of the study [13–16]. Several sources were used to generate a pool of questions relevant to the study objectives [13–16]. The questions were tabled and reviewed by the research team to combine concepts and to remove duplicates if any. The questionnaire was administered in English, since English is the official language of education for pharmacists in the United Arab Emirates.

To ensure face and content validity, the first draft of the questionnaire was evaluated by six independent academics with previous experience in pharmacy practice and education. They informed the research team if the items in the questionnaire were not straightforward or difficult to understand. Comments and feedback provided were considered by the research team and then incorporated where appropriate to develop the final version of the questionnaire. Finally, the research team revised the items as necessary to make them concise and fit online administration.

The final version of the questionnaire was organized into six main sections addressing different topics of interest. The *first section* included items to collect demographic data. The *second section* included items aimed to assess participants’ current epidemics/pandemics and COVID-19 knowledge. The *third section* assessed the participants’ perspective of the role of the pharmacist during epidemics and pandemics and the new coronavirus pandemic. The *fourth section* included items aimed to evaluate participants’ perspectives of the role of the pharmacy educators/educational institutes in preparing future pharmacists to deal with epidemics and pandemics, with a specific focus on the coronavirus pandemic. The *fifth section* included items aimed to assess participants’ perspectives of the role of the health authorities in preparing future pharmacists to deal with epidemics and pandemics, again using the current coronavirus pandemic as a case in point. The *sixth section* assessed the barriers that would hinder pharmacists from performing their duties optimally. The details of each are available in Appendix 1.

### Survey implementation

Study participants were recruited mainly through social media (Facebook and WhatsApp); those willing to consider participation could open a link to initially view ethics committee approval on study conduction and complete the survey. The study’s inclusion criteria of the potential participants were as follows: UAE residents, know the English Language, a licensed pharmacist or pharmacy student. Thus, all the participants have a pharmacy background. The participants were asked about these inclusion criteria at the beginning of the questionnaire and were noted to only complete the questionnaire if they met these requirements. At the end of the data collection phase, the correct answers for the knowledge section of the survey were made available to the participants (as informed in the information section) to improve their knowledge about coronavirus prevention, symptoms, and proposed treatment.

### Sample size

Based on the number of licensed pharmacists in the UAE, sample size calculation using a margin of error of 5%, confidence level of 95%, and response distribution of 50%, a minimum sample size of 375 participants is needed.

For pharmacy students, a sample size calculation was performed using the following formula: *n* = *P* × (100−*P*) × *z*^2^/*d*^2^. Where *P* is the anticipated prevalence (prevalence of knowledge here), d is the desired precision, z is the appropriate value from the normal distribution for the desired confidence. Using 95% confidence level, 5% precision level, and 50% anticipated prevalence of inappropriate knowledge (this conservative prevalence value results in the highest possible sample size that can be used in this study, as no previous studies in this area were found to indicate other prevalence of inappropriate knowledge), a sample size of 375 was considered representative of this sampling frame.

### Statistical analysis

Data were analyzed using the Statistical Package for the Social Science (SPSS) version 22 (SPSS Inc., Chicago, IL, USA). The descriptive analysis was undertaken using mean and standard deviations for continuous variables and percentage for qualitative variables. The Shapiro–Wilk test was used to examine if the variables are normally distributed (with *P*-value ≥ 0.05 indicating a normally distributed continuous variable). Correlations and group differences between students and pharmacists were tested using the chi-square test.

## Results

### Demographic characteristics

Although the minimum sample size (375 participants) was reached before the pre-planned study period (one month), the questionnaire was open for the participants to complete, hence reaching the sample size of 869. More than 70% of the participants were aged between 18 and 30 years. Most participants were females (76.2%), and almost 70% of the participants were not married/single. Nearly half of them (42.9%) have a bachelor’s in pharmacy degree B.Pharm (5-year program)/Pharm D (6-year program). Pharmacy students made up 48.6% of the sample, while the rest ranged between pharmacy owners (who have a bachelor’s degree in pharmacy), community pharmacists, hospital pharmacists, pharmacy trainees, and academics. Most of the participants lived in Sharjah. Participants who were not students had recently graduated from university (1–5 years ago, 21.0%), with 1–5 years of experience (20.2%). One-third of the participants did not attend any educational workshop last year (25.2%), while few attended five or more workshops (23.2%), as shown in Table 1.

**Table 1:** Demographic characteristics of the study sample at baseline (*n* = 869)

Parameters	*n* (%)
Age, *n* (%)	
• 18–24 • 25–30 • 31–35 • 36–40 • 41–45 • Above 45	493 (56.7) 129 (14.8) 77 (8.9) 58 (6.7) 55 (6.3) 57 (6.6)
Gender, *n* (%)	
• Female • Male	662 (76.2) 207 (23.8)
Marital status, *n* (%)	
• Married • None-married (single, divorced, or widowed)	267 (30.8) 602 (69.3)
Educational level, *n* (%)	
• Undergraduate studies • Diploma • B. Pharm • Pharm. D • Masters • PhD	319 (36.7) 26 (3.0) 334 (38.4) 39 (4.5) 126 (14.5) 25 (2.9)
Employment, *n* (%)	
• Pharmacy owner • Community pharmacist • Hospital pharmacist • Pharmacy trainee • Faculty member • Trainee	17 (2.0) 124 (14.3) 162 (18.6) 71 (8.2) 72 (8.3) 422 (48.6)
Living area, *n* (%)	
• Abu Dhabi • Dubai • Sharjah • Ajman • Umm Al Quwain • Ras Al Khaimah • Fujairah • Outside the UAE	189 (21.7) 147 (16.9) 371 (42.7) 82 (9.4) 7 (0.8) 38 (4.4) 13 (1.5) 22 (2.6)
Graduation years, *n* (%)	
• Current graduated • 1–5 years ago • 6–15 years ago • 16–25 years ago • More than 25 years ago	425 (48.9) 182 (21.0) 151 (17.4) 69 (7.9) 42 (4.8)
Years of experience, *n* (%)	
• No experience • 1–5 years • 6–10 years • 11–15 years • 16–20 years • 21–25 years • More than 25 years	456 (52.5) 176 (20.2) 80 (9.2) 60 (6.9) 38 (4.4) 27 (3.1) 32 (3.7)
The number of attended educational workshop in the last year, *n* (%)	
• 0 • 1 • 2 • 3 • 4 • 5 • More than 5	219 (25.2) 106 (12.2) 153 (17.6) 122 (14.0) 68 (7.8) 42 (4.8) 159 (18.4)

**Fig. 1:**
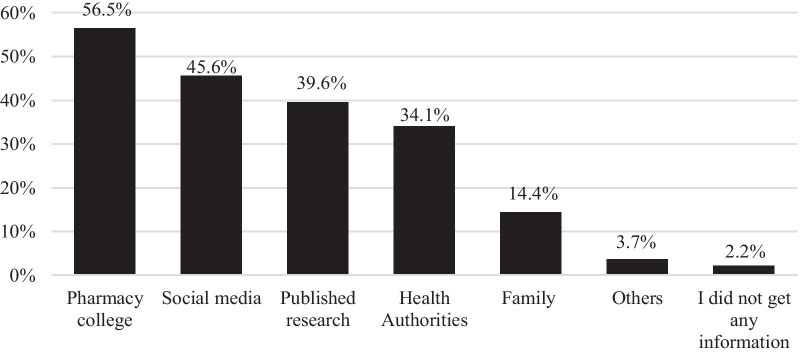
Participants' sources of information to read about the terms "epidemics" or "pandemics"

About half of the study participants (57.5%) reported that they got enough education previously about epidemic/pandemic topics. The main sources of information reported to read about the epidemic/pandemic, in general, were the college of pharmacy (56.5%) and social media (45.6%), as shown in Fig. 1. Participants who have access to pharmacy college can be students, staff, academic, research or teaching assistants.

**Fig. 2:**
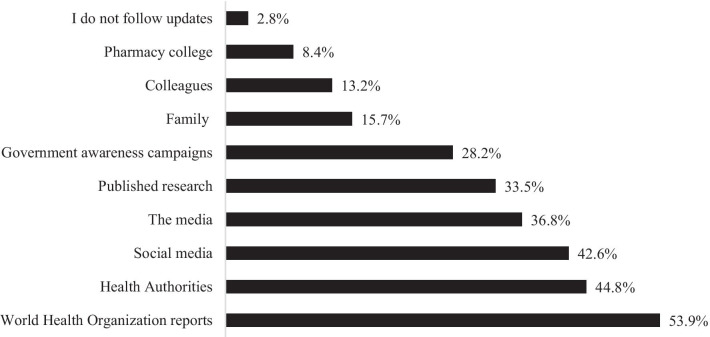
Participants' sources of information to get the latest updates on COVID-19 treatment

As shown in Fig. 2, the majority (88.3%) reported that they currently followed the latest coronavirus updates on treatments quite closely. They updated their information mostly from the World Health Organization reports, health authorities, and social media indicating the use of Facebook, WhatsApp, Twitter, etc., which is different from media, such as television and radio.

### The knowledge regarding COVID-19 among pharmacists/pharmacy students

**Fig. 3:**
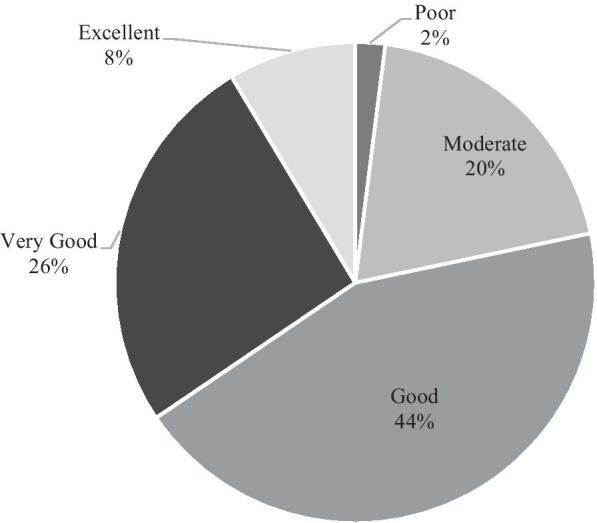
Participants' assessment of their current knowledge about COVID-19 five months after the outbreak

Participants were asked to assess their current knowledge about COVID-19 after five months of the outbreak using a 5-point Likert scale (poor, moderate, good, very good, and excellent knowledge), and about 70% of the participants had good/very good knowledge (Fig. 3).

When assessing knowledge about coronavirus pandemic, the majority of responses demonstrated correct answers to the items except for the item: “*Using steroids do not increase vulnerability to coronavirus infection*”, 63.7% answered it wrongly, followed by “*Hydroxychloroquine has not been used as a preventative therapy against coronavirus infection*”, “*Oseltamivir has not been used in the management of coronavirus infection cases*”, “*You need to keep a distance of at least 3 m (10 feet) when counselling patients during a pandemic*”, and “*The highest population risk category for people acquiring the coronavirus infection are: elderly (> 65), immunocompromised people or children under the age of 9*”. Children under the age of 9 are not among the highest risk people to acquire the coronavirus (Table 2), yet 90.7% of participants chose this statement as 'True'.

**Table 2:** Assessment of study participants (pharmacists and students) knowledge about coronavirus pandemic (*n* = 869)

Statements	Correct answer, *n* (%)			
	Total *n* = 869	Students *n* = 382	Pharmacists *n* = 404	*P*-value^#^
One way of transmission of coronavirus is respiratory droplets from person to person among close contacts	623 (93.8)	301 (93.2)	321 (94.4)	0.802
Coronavirus can be transmitted after touching surfaces that were contaminated with the virus	607 (91.3)	298 (92.0)	308 (90.6)	0.796
Non-steroidal anti-inflammatory drugs such as ibuprofen can decrease the risk of complications when used during viral infections	399 (60.3)	145 (45.0)	253 (74.6)	< 0.001*
Fevers/ Dry cough/shortness of breath are associated with coronavirus	642 (97.0)	313 (97.5)	328 (96.5)	0.727
Muscle aches and gastrointestinal symptoms (nausea/vomiting/diarrhea) are not associated with coronavirus	384 (57.7)	140 (43.2)	244 (71.8)	< 0.001*
Hand washing with soap and water for 20 s is enough to clean the hands and protect from spreading the infection	548 (82.7)	258 (79.9)	289 (85.3)	0.045*
Using steroids does not increase vulnerability to Coronavirus infection	241 (36.3)	81 (25.1)	160 (47.2)	< 0.001*
In general, the use of autoimmune disease treatments increases the susceptibility to get infected with the coronavirus	387 (58.3)	164 (50.6)	223 (65.8)	< 0.001*
Hydroxychloroquine has not been used as a preventative therapy against coronavirus infection	256 (38.7)	94 (29.1)	161 (47.8)	< 0.001*
Azithromycin has been used along with hydroxychloroquine in the treatment of the coronavirus infection cases	311 (46.8)	97 (29.9)	214 (63.1)	< 0.001*
Oseltamivir has not been used in the management of coronavirus infection cases	168 (25.3)	55 (17.0)	113 (37.3)	< 0.001*
Protein calorie malnutrition impairs host immunity (particularly the T-cell system) resulting in increased opportunistic infections	317 (47.0)	145 (44.9)	171 (50.9)	0.022*
Patients should eat food that contains Vitamin C and D to boost their immunity	620 (93.5)	298 (92.0)	321 (95.0)	0.003*
Eating food such as mushrooms and garlic is beneficial for the immune system	487 (73.7)	233 (71.9)	253 (75.3)	0.005*
Exercise causes antibodies and white blood cells to circulate in the body more rapidly detecting infections at an early stage	441 (66.7)	218 (67.3)	222 (66.1)	0.009*
The short-term rise in body temperature during and right after exercise increases bacterial growth, which will lower the body's ability to fight the infection	388 (58.7)	171 (52.9)	216 (64.1)	0.002*
Not smoking and decreasing stress help support the immune system	624 (94.5)	303 (94.1)	320 (95.0)	0.751
Sunlight activates T-helper cells hence boosts immunity	399 (60.5)	175 (54.3)	224 (66.5)	< 0.001*
You need to keep a distance of at least 3 m (10 feet) when counseling patients during a pandemic	168 (25.4)	68 (21.1)	100 (29.7)	0.001*
The highest risk patients in acquiring the coronavirus infection are elderly (> 65), immune-compromised and children under the age of 9	61 (9.3)	29 (9.0)	3 2(9.5)	0.062

*significant at ≤ 0.05 significance level

^#^Using Chi-square test, *significant at ≤ 0.05 significance level

For the rest of the knowledge items comparing pharmacists and student participants, pharmacists knew that non-steroidal anti-inflammatory drugs such as ibuprofen might theoretically increase the risk of complications when used during viral infections (p = 0.001) and that muscle aches and gastrointestinal symptoms (nausea/vomiting/diarrhoea) could be associated with coronavirus infections (p < 0.001). Similar significance was found for the items stating that “*Steroids may increase the susceptibility to coronavirus infection in some case*s”, “*Generally, the use of autoimmune disease treatments increases the susceptibility to acquire the coronavirus infection*”, “*Hydroxychloroquine has not been trialled as a preventative therapy against coronavirus infection*”, “*Azithromycin has been trialled along with Hydroxychloroquine in the treatment of the coronavirus infection*”, and “*Sunlight activates T-helper cells hence boosts immunity*”, (*p* < 0.001 for all).

### The role of the pharmacists

Table 3 shows the study participants’ perceptions about the role of pharmacists to deal with epidemics/pandemics generally and COVID-19 specifically. More than 85.0% strongly agreed/agreed that they have a major role in the management of pandemics/epidemics through their pharmacies and that it is their responsibility to ensure the availability of key medications (86.9%). The majority (87.7%) also believed that it was their role to counsel people about coronavirus infection and how to reduce the transmission and the spread of disease. More than 90.0% strongly agreed/agreed that they were responsible for their personal safety by avoiding close contact with all patients. Similar findings were reported with the other items in Table 3 except with the following item: “*You provide Hydroxychloroquine to patients who need it even if they do not have a prescription*”, where the majority (70.0%) disagreed/strongly disagreed with this statement.

**Table 3:** Study participants perceptions about the role of pharmacists to deal with epidemics/pandemics generally and COVID-19 (*n* = 869)

Statements	Strongly disagree, *n* (%)	Disagree, *n* (%)	Neutral, *n* (%)	Agree, *n* (%)	Strongly agree, *n* (%)
The pharmacist has a major role in the management of pandemics/epidemics	16 (2.6)	14 (2.2)	57 (9.1)	271 (43.4)	267 (42.7)
It is the pharmacist role to ensure the availability of relevant medicines/products	13 (2.1)	11 (1.8)	58 (9.3)	273 (43.6)	271 (43.3)
It is the pharmacist role to counsel people about coronavirus infection and how to reduce the transmission and the spread of disease	8 (1.3)	8 (1.3)	61 (9.8)	273 (43.7)	275 (44.0)
The pharmacist ensures his/her personal safety by wearing gloves and masks and avoid close contact with patients	6 (1.0)	3 (0.5)	31 (5.0)	187 (30.0)	369 (63.6)
If you suspect someone may have coronavirus, you know how to seek immediate medical attention	10 (1.6)	23 (3.7)	100 (16.0)	252 (40.3)	240 (38.4)
You provide Hydroxychloroquine to patients who need it even if they do not have a prescription	226 (36.2)	211 (33.8)	85 (13.6)	70 (11.2)	32 (5.1)
Pharmacies should be allowed to send medications to their patients home during the coronavirus pandemic when needed	14 (2.2)	21 (3.4)	60 (9.6)	284 (45.4)	246 (39.4)
You should be allowed to sell medications for coronavirus management via drive-through	49 (7.9)	79 (12.7)	135 (21.6)	219 (35.1)	142 (22.8)

### The role of the pharmacy facilities

Table 4 shows the role of the colleges of pharmacy in preparing pharmacists for their responsibilities during epidemics/pandemics, and specifically the coronavirus pandemic. The majority of participants (68.0%) agreed/strongly agreed the college has a role in preparing the pharmacist to deal with any epidemics/pandemics. Moreover, most participants stated that the college should add an epidemics/pandemics management course, online lectures and webinars, online educational workshops on the pandemic coronavirus, and should provide online information resources (e.g., summaries of current research studies) for the pandemic.

**Table 4:** Perceptions of participants (*n* = 869) regarding role of the colleges of pharmacy in preparing pharmacists for their responsibilities during epidemics/pandemics, and specifically the coronavirus pandemic

Statements	Strongly disagree, *n* (%)	Disagree, *n* (%)	Neutral, *n* (%)	Agree, *n* (%)	Strongly agree, *n* (%)
The college has a role in preparing you to deal with any epidemics/pandemics	33 (5.2)	42 (6.6)	127 (20.1)	230 (36.4)	200 (31.6)
The college should add an epidemics/pandemics management course	31 (4.9)	20 (3.71)	86 (13.6)	269 (42.6)	226 (35.8)
The college should provide you with online lectures and webinars as a student or alumni	18 (2.8)	11 (1.7)	99 (15.7)	286 (45.3)	218 (34.5)
The college should provide online educational workshops on the pandemic coronavirus	21 (3.3)	13 (2.1)	68 (10.8)	301 (47.7)	228 (36.1)
The college should provide online information resources (e.g., summaries of current research studies) on the pandemic coronavirus	15 (2.4)	10 (1.6)	58 (9.2)	282 (44.7)	266 (42.2)

### The role health authorities

Regarding the role of the health authorities, more than 80% of the participants agreed/strongly agreed that the health authorities have a role in preparing pharmacists to deal with epidemics/pandemics such as the coronavirus pandemic and that they should be sending them knowledge emails (85.5%) and provide online educational workshops (84.2%) on the coronavirus pandemic. Many of the participants agreed/strongly agreed that the health authorities should monitor the availability of the medications used to manage the coronavirus in the pharmacies (89.9%). More than 85.0% of the participants agreed/strongly agreed that the health authorities and the pharmacy colleges should join forces to produce one educational module for the management of the coronavirus pandemic (Table 5).

**Table 5:** Perceptions of study participants (*n* = 896) of the current role of the health authorities in combating epidemics/pandemics and specifically the coronavirus infection

Statements	Strongly Disagree, *n* (%)	Disagree, *n* (%)	Neutral, *n* (%)	Agree, *n* (%)	Strongly Agree, *n* (%)
The health authorities have a role in preparing you to deal with epidemics/ pandemics, such as coronavirus	20 (3.1)	11 (1.7)	75 (11.87)	276 (43.6)	250 (39.5)
The health authorities should be sending you awareness emails on the current coronavirus pandemic	14 (2.2)	8 (1.2)	69 (10.9)	281 (44.5)	259 (41.0)
The health authorities should provide online educational workshops on the pandemic coronavirus	16 (2.5)	8 (1.2)	75 (11.8)	275 (43.5)	257 (40.7)
The health authorities should monitor the availability of the medications used in the management of the coronavirus in pharmacies	14 (2.2)	5 (0.79)	44 (6.9)	247 (39.2)	320 (50.7)
The colleges of pharmacy and health authorities should join forces to produce one educational module for the management of the coronavirus pandemic	16 (2.54)	5 (0.79)	70 (11.0)	262 (41.5)	278 (44.0)

### Barriers faced by the pharmacists

Almost 62% of participants reported their fear and anxiety of acquiring the coronavirus infection. Only 21.3% are less willing to work during the pandemic (Table 6).

**Table 6:** Participants' responses about their level of personal fear and anxieties about the COVID-19 pandemic

Statements	Strongly disagree*, n (%)*	Disagree, *n (%)*	Neutral, *n (%)*	Agree, *n (%)*	Strongly agree, *n (%)*	Not applicable, *n (%)*
Working in a pharmacy increase your concern about getting the coronavirus infection	16 (2.6)	27 (4.4)	110 (17.9)	243 (39.6)	140 (22.8)	77(12.6)
You are less willing to work during the coronavirus pandemic	116 (19.0)	185 (30.2)	125 (20.4)	91 (14.9)	39 (6.4)	56 (9.2)
If you have symptoms, you are afraid to let your employer know for the fear of losing your job	259 (42.4)	165 (27.0)	48 (7.9)	59 (9.7)	21 (3.4)	59 (9.7)
If you are/were a pharmacy owner, you are concerned from not getting enough financial benefits, during the coronavirus pandemic	57 (9.3)	86 (14.1)	150 (24.6)	151 (24.8)	53 (8.7)	113 (18.5)
You think that going out for work poses a risk to the health of your family members	12 (2.9)	20 (3.3)	99 (16.2)	241 (39.4)	189 (30.9)	51 (8.3)
You are afraid of getting depression/posttraumatic stress symptoms/anxiety as a consequence of working during the coronavirus pandemic	57 (9.3)	129 (21.1)	130 (21.3)	165 (27.0)	66 (10.8)	64 (10.5)
If you receive bonus pay, that would increase your willingness to work during coronavirus pandemic	23 (3.8)	61 (10.0)	150 (24.5)	188 (30.7)	123 (20.1)	68 (11.1)
You prefer the existing of preventive measures such as screening the employees every now and then	11 (1.8)	13 (2.1)	77 (12.6)	245 (40.2)	214 (35.1)	50 (8.2)
Every pharmacy should offer phone/online support from a psychologist and teach stress management techniques	18 (2.9)	13 (2.1)	105 (17.2)	254 (41.5)	176 (28.8)	46 (7.5)
You prefer working for 2 weeks then home self-isolation for two weeks, because it will decrease your fear of acquiring the coronavirus infection	39 (6.4)	80 (13.2)	145 (23.8)	184 (30.3)	89 (14.6)	71 (11.7)

## Discussion

The role of pharmacists is vital and complementary to the role of other healthcare workers in handling the COVID-19 global crisis. Community pharmacists are highly accessible and feasible to be reached by patients, being a reliable source of information regarding COVID-19 as well as providing patients with medication counselling and other needed services [17, 18]. Pharmaceutical associations and facilities should implement new strategies to help pharmacists fulfil their roles, helping them overcome the obstacles and barriers in such challenging circumstances [19, 20].

Through this study, we investigated the pharmacist's preparedness, knowledge regarding COVID-19, role of pharmacists during the current pandemic, role of pharmacy facilities and health authorities in managing COVID-19, and barriers that hindered pharmacists from performing their duties optimally. The majority of participants agreed on the importance of their role during this pandemic, similar to other studies conducted worldwide assessing pharmacists' contribution to this pandemic [17, 20–22].

### The knowledge regarding COVID-19 among pharmacists/pharmacy students

The level of knowledge that pharmacists have regarding the COVID-19 is very important for supporting patients in this crisis, as it can affect their clinical practice. Our study findings reported that almost two-thirds of them had good/very good knowledge. Also, the majority closely followed the latest coronavirus updates on treatments and updated their information mostly from the WHO reports, health authorities, and social media, similar to findings reported by other pharmacists in the MENA region [23, 24]. It is crucial that pharmacists worldwide stay updated and use a reliable source of information during a pandemic. In response to the COVID-19 crisis, many organizations, implemented the use of programs that maintained and improved the knowledge of pharmacists regarding the COVID-19 pandemic. Free access to courses about COVID-19 was granted to pharmacists, including the prevention and treatment strategies and patient safety concerns.

### The role of the pharmacists

As for the role of pharmacists, it is expressed in the importance of providing the patients with needed medications, counselling the patients about coronavirus infection, and guiding them on how to reduce the transmission and the spread of the virus. Their role in controlling infections and public safety is a very important aspect of good pharmacy practice, which aims to provide patients with the most appropriate health care [25, 26].

### The role of pharmacy facilities and health authorities

Pharmaceutical associations and organizations at national levels should clearly define pharmacists' roles and support the needed policies, laws, and strategies to help them fulfil their roles during such pandemics [19]. The International Pharmaceutical Federation (FIP) released in March 2020 a guideline affirming the information needed for pharmacists and pharmacy workforces to help them in dealing with the COVID-19 outbreak clarifying their own responsibilities during the COVID-19 pandemic [27]. In close by countries, such as Jordan, the Food and Drug Administration (JFDA) allowed community pharmacists and hospitals to deliver medications to patients as an effective service to reduce the risk of viral transmission, which is not allowed in normal circumstances [20]. This is a positive example for implementing new effective policies that attributed effectively to the role of pharmacists during the COVID-19. In addition, the role of health authorities and educational institutions is essential in enhancing the pharmacists' knowledge about COVID-19, preparing them to deliver their roles during pandemics. According to the study findings, most pharmacists highlighted the positive role played by the health authorities in the UAE, which is similar to what was shown in previously published findings [19, 20].

Most pharmacy students stressed the need to add a management course/study unit within the curriculum related to pandemics. The fact that a significant difference was found between pharmacists' knowledge compared to students’ knowledge regarding the pandemic, adds to the importance of adding such focused courses to the current pharmacy curriculum. The need for educational and health authorities to prepare pharmacy students and pharmacists for emergency events, such as pandemics, is crucial to ensure an effective role of pharmacists in the management of pandemics. Most pharmacists agreed on the need for health authorities to prepare them for such events by sending awareness emails and providing online educational workshops. Hence, to ensure an effective role of pharmacists in the management of pandemics, pharmacists need earlier training and follow-up to prepare them in advance for such circumstances. In addition, the health authorities should monitor the availability of medications used in the management of the coronavirus in pharmacies, as stated by the study participants.

Different medications have been mentioned in the management of COVID-19, for example, Chloroquine or Hydroxychloroquine, that have been used by specialists in hospitals in addition to ventilatory support for patients with severe respiratory distress symptoms. Nevertheless, some of the participants acknowledged the use of neither Chloroquine/Hydroxychloroquine as a preventative therapy nor Oseltamivir in managing coronavirus infection cases. At present, there are more than 140 clinical trials to test the efficacy of these medications alone or in combination with other medications to explore their use as treatment/prevention options for COVID-19 [28, 29], as well as several speculations have been going on regarding the use of non-steroidal anti-inflammatory medications (NSAIDs), such as Ibuprofen as antipyretic in the treatment of coronavirus infection. The majority of the study participants agreed that using NSAIDs such as Ibuprofen would increase the risk of COVID-19 complications. As the Pharmacovigilance Risk Assessment Committee (PRAC) has stated the worsening of COVID-19 infection in patients using Ibuprofen/Ketoprofen, extreme caution and medical supervision are needed on the usage of NSAIDs [30].

### Barriers faced by the pharmacists

As for the barriers, pharmacists reported their responsibility for their personal safety by avoiding close contact with all patients, and in addition to their personal fear and anxieties about the coronavirus pandemic, reporting that working in community pharmacies increased their concern about acquiring the coronavirus infection. Participants' opinions about their level of personal fear and anxieties about the COVID-19 pandemic mentioned in Table 6 impede the pharmacists from acting to their full potential during such emergencies. Similarly, this was reported by other pharmacists in other countries [31, 32].

## Conclusion

The majority of pharmacists and pharmacy students reported that they have a major role in managing pandemics delivered through the community pharmacies and that it is their role to ensure the availability of key medications. Hence, the policymakers and health authorities need to prepare and train the pharmacists in advance, providing them with the needed professional mental support and help which is crucial to overcome their stresses and anxieties.

## Data Availability

Data available on request due to privacy/ethical restrictions.

## Appendix 1

The final version of the questionnaire was organized into six main sections addressing different topics of interest. The *first section* included items to collect demographic data. The *second section* included items aimed at assessing potential participants' current epidemics/pandemics and COVID-19 knowledge; here, potential participating pharmacists were asked about how much they know about the coronavirus, including a) symptoms, b) modes of transmission, c) how to prevent the transmission and the spread of the virus, and d) awareness about where to access the latest coronavirus updates regarding treatment. For each of these sections, there were several detailed items, e.g., participants, when asked, for example, were probed about the modes of coronavirus transmission, such as if the virus can be transmitted from the respiratory droplets from person to person among close contacts and after touching surfaces that were contaminated by the virus [33]. Other questions investigated participants' knowledge regarding the highest risk patients in addition to the symptoms that are associated with coronaviruses, such as fever, dry cough, shortness of breath, muscle aches and gastrointestinal symptoms [33]. The latest coronavirus updates on treatment were questioned, for example, if Hydroxychloroquine alone or in combination with Azithromycin has been used as a preventive therapy against coronavirus infection or if Oseltamivir is being used in the management of the coronavirus infection [33–37]. Another question assessed the knowledge concerning using non-steroidal anti-inflammatory drugs, such as Ibuprofen when there is an infection [38, 39]. Other questions in the treatment section were whether using some drugs (glucocorticoids and autoimmune disease medications) increase the susceptibility to get infected with the coronavirus [40, 41]. Participants' knowledge of how to prevent the transmission and the spread of the virus was assessed in-depth as well, such as if washing hands with soap and water for 20 seconds would be enough to clean the hands, also the least distance that should be kept while counselling patients (2 m/6 feet) [33, 42, 43]. Two questions were about exercise, the first one asked if exercise causes antibodies and white blood cells to circulate more rapidly in the body, therefore, detecting and combating infections at an early stage, and the second question asked if the brief rise in body temperature during and after exercise prevents bacterial growth, thus increases the body's ability to fight the infection [14, 44]. The rest of the questions were about the immune system and ways to boost it, to combat the viral infection [14, 33, 45, 46]. For example, participants were asked if protein-calorie malnutrition increases the opportunistic infection [47, 48]; and the role of garlic, mushrooms, vitamin C and D was also questioned. Not smoking and the importance of decreasing stress to boost immunity were also questioned [49–51]. And finally, the effect of sunlight in activating the T-helper cells to boost immunity was questioned [52]. Participants were asked to provide their email to receive the answers for the knowledge questions once the study was completed if they wished to receive this information. Potential participants were also given a choice to view the correct answers via a Facebook page designed for this study. All items in this section required a True/False/Not sure response.

The *third section* concentrated on the potential participants' perspective of the role of the pharmacist during epidemics and pandemics and the new coronavirus pandemic; it included eight closed-ended questions. The participants' responses to this section used a Likert scale answer, i.e., "Strongly agree", "Agree", "Neutral", "Disagree", or "Strongly disagree".

The *fourth section* included items aimed at assessing participants' perspectives of the role of the pharmacy educators/educational institutes in preparing future pharmacists to deal with epidemics and pandemics, with a specific focus on the coronavirus pandemic. There were several detailed items, e.g., if faculties should add a course to cover information on epidemics/pandemics and the role of the pharmacist during such times, the need for online lectures and webinars provision for students and alumni, online educational workshops provision on the current coronavirus pandemic, and provision of online information resources discussing international and local needs and findings (e.g., summary of local and international research outputs) on the coronavirus pandemic. The *fifth section* included items aimed at assessing participants' perspectives of the role of the health authorities in preparing future pharmacists to deal with epidemics and pandemics, again using the current coronavirus pandemic as a case in point. Here participants were asked if the health authorities should be sending registered pharmacists awareness emails clarifying important issues regarding the coronavirus pandemic or be providing online educational workshops, online training resources or patient education materials for distribution. Participants' were also asked if the health authorities should have a role in monitoring the availability of the medications used in the management of the coronavirus infection in community pharmacies (e.g., to counter disease-mongering via social media and resultant hoarding response of consumers). And lastly, if the faculties of pharmacy nationally and the health authorities should join forces and produce one educational module for the management of the pandemic coronavirus.

Section four and five included five closed-ended questions, and the participants' responses to these sections were conveyed using a Likert scale, i.e., "Strongly agree", "Agree", "Neutral", "Disagree", or "Strongly disagree".

The *sixth section* concentrated on the barriers that would hinder pharmacists from performing their duties optimally. There were several detailed items, e.g., if working in a pharmacy would increase the participants’ concern regarding getting infected, if they are less willing to work during the coronavirus pandemic, if they have symptoms, they would be afraid to tell their employer for the fear of losing their job, if they were a pharmacy owner, they are concerned from not getting enough financial benefits, during coronavirus pandemic, if they think that going out for work poses a risk to the health of their family members, if they are afraid of getting depression/posttraumatic stress symptoms/anxiety as a consequence of working during the coronavirus pandemic, if they receive bonus pay, that would increase their willingness to work during coronavirus pandemic, if they prefer the existing of preventive measures such as screening the employees every now and then, if every pharmacy should offer phone/online support from a psychologist and teach stress management techniques, and if they prefer working for two weeks then home self-isolation for two weeks, because it will decrease their fear of acquiring coronavirus infection.
